# Carotenoid-Producing *Paracoccus aurantius* sp. nov., Isolated from the West Coast of Dokdo Island, Republic of Korea

**DOI:** 10.4014/jmb.2404.04053

**Published:** 2024-08-09

**Authors:** Chi Young Hwang, Eui-Sang Cho, Eun Hee Bae, Dong-Hyun Jung, Myung-Ji Seo

**Affiliations:** 1 Department of Bioengineering and Nano-Bioengineering, Incheon National University, Incheon 22012, Republic of Korea; 2 Biotechnology Institute, University of Minnesota, St. Paul, MN 55108, USA; 3 Climate Change and Environmental Biology Research Division, National Institute of Biological Resources, Incheon 22689, Republic of Korea; 4 Division of Food and Nutrition, Chonnam National University, Gwangju 61186, Republic of Korea; 5 Division of Bioengineering, Incheon National University, Incheon 22012, Republic of Korea; 6 Research Center for Bio Materials & Process Development, Incheon National University, Incheon 22012, Republic of Korea

**Keywords:** *Paracoccus*, Dokdo Island, polyphasic taxonomy, carotenoid

## Abstract

In this study, a novel species within the genus *Paracoccus* was isolated from the coastal soil of Dokdo (Seodo) Island and investigated. We elucidated the novel species, designated MBLB3053^T^, through genomic analysis of novel functional microbial resources. Cells were gram-negative, non-motile, and coccoid, and the colony was light orange in color. Phylogenetic analysis based on the 16S rRNA gene showed that strain MBLB3053^T^ was related to the genus *Paracoccus*, with 98.5% similarity to *Paracoccus aestuariivivens*. Comparative genome analysis also revealed the strain to be a novel species of the genus *Paracoccus* by average nucleotide identity and in silico DNA-DNA hybridization values. Through secondary metabolite analysis, terpene biosynthetic gene clusters associated with carotenoid biosynthesis were found in strain MBLB3053^T^. Using high-performance liquid chromatography, strain MBLB3053^T^ was confirmed to produce carotenoids, including all-*trans*-astaxanthin, by comparison to the standard compound. Notably, the isolate was also confirmed to produce carotenoids that other closely related species did not produce. Based on this comprehensive polyphasic taxonomy, strain MBLB3053^T^ represents a novel species within the genus *Paracoccus*, for which the name *Paracoccus aurantius* sp. nov is proposed. The type strain was MBL3053T (=KCTC 8269^T^ =JCM 36634^T^). These findings support the research and resource value of this novel species, which was isolated from the Dokdo environmental microbiome.

## Introduction

The family *Paracoccaceae* (named as *Rhodobacteraceae*), within the class Alphaproteobacteria, comprises gram-negative bacteria found in diverse environments, and holds a significant position within the microbial community [1]. Members of the class Alphaproteobacteria, including *Paracoccaceae*, are known for their remarkable adaptability and versatile metabolic capabilities [1, 2]. They are frequently encountered in various ecological niches, ranging from soil and aquatic ecosystems, to symbiotic associations with plants. The genus *Paracoccus*, as the type genus of the family *Paracoccaceae*, serves as a significant representative within this taxonomic group. Currently, a total of 83 species have been validly published in the List of Prokaryotic names with Standing in Nomenclature (LPSN, https://lpsn.dsmz.de/), and the type species is *P. denitrificans* [3].

The bacteria belonging to the genus *Paracoccus* are known for their various beneficial properties, including nitrogen cycling and atmospheric nitrate mineralization [4, 5]. Notably, certain members of the genus *Paracoccus* have been reported to produce carotenoids, such as *P. marcusii* [6], *P. carotinifaciens* [7], *P. zeaxanthinifaciens* [8], *P. haeundaensis* [9], and *P. bogoriensis* [10]. Carotenoids are pigments with important roles in natural environments; they can impact the diversity and stability of ecosystems while affecting various biological processes. The carotenoid biosynthesis gene clusters were identified as astaxanthin biosynthesis enzymes from *P. haeundaensis* [11]. A sub-chronic toxicity evaluation using carotenoid extracts from *P. carotinifaciens* suggests a potent antioxidant associated with numerous potential health benefits [12]. In addition, *Paracoccus* sp. LL1 is associated with the co-production of carotenoids and biodegradable plastic through bioconversion [13].

As a well-known, representative island of the Republic of Korea, Dokdo possesses a preserved, natural ecosystem imbued with high ecological and academic value along with its distinctiveness [14]. Moreover, the further discovery of indigenous microorganisms derived from the Dokdo environmental microbiome is anticipated to contribute to expanding the biodiversity of the Korean Peninsula. In this study, we aimed to elucidate a novel species from Dokdo through genomic analysis of novel functional microbial resources.

## Materials and Methods

### Isolation and Culture Conditions

Strain MBLB3053^T^ was isolated from a soil sample collected off the western coast of Dokdo Island, Republic of Korea (37°14'29.5"N 131°52'00.2"E) in 2022. The sea soil sample weighed 1.0 g and was suspended in 10 ml of 0.85% (w/v) NaCl solution. Subsequently, the sample was serially diluted in fresh Reasoner’s 2A (R2A) broth (KisanBio, Republic of Korea), and aliquots of 100 μl from the diluted samples were then evenly spread on R2A agar plates. Each plate was incubated at 30°C for a period of one week. After incubation, the isolated colonies were sub-cultured by streaking them onto fresh R2A agar plates at least three times to ensure pure cultures. Finally, one bright-orange colony of the isolate, designated as MBLB3053^T^, was selected for further experiments. Strain MBLB3053^T^ was experientially cultured on R2A agar plates at 30°C for 24 h under aerobic conditions and then preserved for the long term at -80°C in a solution containing 20% (w/v) glycerol stock. For taxonomic analysis, the reference strains *P. aestuariivivens* KCTC 52214^T^, *P. litorisediminis* KCTC 52978^T^, and *P. sordidisoli* KCTC 42938^T^ were purchased from the Korean Collection for Type Cultures (KCTC) [15-17].

### Phylogenetic Analysis

The genomic DNA (gDNA) of strain MBLB3053^T^ was extracted using the LaboPass Genomic DNA Isolation Kit (Cosmogenetech, Republic of Korea). A fragment of 16S rRNA gene was amplified through polymerase chain reaction (PCR) using universal bacterial primers, including 27F (5'-AGAGTTTGATCMTGGCTCAG-3') and 1492R (5'-TACGGYTACCTTGTTACGACTT-3'). The PCR product was subsequently sequenced by Macrogen Co., Ltd. (Republic of Korea) and the obtained partial sequences were analyzed using BioEdit v7.2.6.1. The 16S rRNA gene sequence of strain MBLB3053^T^ was deposited in the National Center for Biotechnology Information (NCBI) database under the accession number PP951778. The sequences of closely related species of strain MBLB3053^T^ were obtained from the EzBioCloud server (http://www.ezbiocloud.net/) [18]. To explore the evolutionary relationship between strain MBLB3053^T^ and closely related species, all 16S rRNA gene sequences were aligned via the CLUSTALW multiple alignment program [19]. Then, using the 16S rRNA gene a phylogenetic tree was constructed using the bootstrap method with 1,000 replicates. The three algorithms and methods of maximum-likelihood (ML) [20], neighbor-joining (NJ) [21], and maximum parsimony (MP) [22] via MEGA X were used [23]. The Kimura two-parameter model was selected as the substitution model [24]. As an outgroup, *Hyphomonas polymorpha* PS728^T^ was employed in the analysis.

### Morphology, Physiological, and Biochemical Analysis

Strain MBLB3053^T^ was cultivated on R2A agar or broth to investigate the traits in this study. Gram staining was assessed using a Gram stain kit according to the manufacturer’s protocols (BioWORLD, USA). Cell morphology was observed by both light microscopy CX 23 (Olympus, Japan) and transmission electron microscopy LIBRA 120 (Carl Zeiss, Germany). The cell motility was examined by dispersal of colonies using stabbed inoculation into a 0.5% semi-solid agar tube [25]. Growth at 4, 10, 15, 20, 25, 30, 37, 40, 45, and 55°C was measured to determine the temperature range for optimal growth. The pH range test was performed using the following buffer solutions: 100 mM CH_3_COOH/CH_3_COONa buffer (pH 4.0–6.0), 100 mM NaH_2_PO_4_/ Na_2_HPO_4_ buffer (pH 7.0–8.0), and 100 mM NaHCO_3_/Na_2_CO_3_ buffer (pH 9.0–10.0). The NaCl range was investigated by supplementing different concentrations of NaCl in R2A broth (0–10.0% at increments of 1.0%). Catalase and oxidase tests were performed as described in a previous report [26]. Casein, starch, carboxymethylcellulose (CMC), Tween 20, 40, and 80 hydrolysis were conducted based on previously reported protocols [27]. H2S formation was monitored using R2A broth containing 0.5% (w/v) sodium thiosulfate with lead acetate paper. Anaerobic growth was examined in an anaerobic gas-generating pouch system by GasPak EZ with an indicator (BD, USA). Antibiotic susceptibility was assessed on R2A plates at 30°C for 2 days using the paper disc method [28]. The following antibiotics were used in this experiment (μg/disc): ampicillin (10), cephalothin (30), erythromycin (25), gentamicin (30), kanamycin (30), lincomycin (15), neomycin (30), norfloxacin (20), novobiocin (10), penicillin G (20 IU), streptomycin (50), and tetracycline (30). Further biochemical properties were investigated using the API 20NE, API ZYM, and API 50 CH test kits, following the manufacturer’s instructions (bioMérieux, France).

### Chemotaxonomic Analysis

Strain MBLB3053^T^ and closely related species (*P. aestuariivivens* KCTC 52214^T^, *P. litorisediminis* KCTC 52978^T^, and *P. sordidisoli* KCTC 42938^T^) were cultivated on R2A plates at 30°C for 2 days for cellular fatty acid analysis. Fatty acids of each strain were analyzed using Agilent 6890 gas chromatography (Agilent, USA) with a cross-linked methyl siloxane column (HP-1; A30 m × 0.320 mm × 0.25 μm) [29]. The profiles were analyzed by Sherlock MIS software version 6.2 according to the TSBA6 database [30]. For isoprenoid quinone analysis, strain MBLB3053^T^ was freeze-dried and isoprenoid quinones were extracted according to the previously described method using YL9100 HPLC (YOUNGIN Chromass, Republic of Korea). [31]. To analyze the polar lipid profiles, cells of strain MBLB3053^T^ were freeze-dried and extraction was performed according to a previously reported method [32]. To detect the polar lipid profiles, two-dimensional thin-layer chromatography (TLC) was performed using 10 × 10 cm silica gel 60 F254 (Merck, Darmstadt, Germany) and visualized by spraying with molybdophosphoric acid, Zinzadze’s reagent, and α-naphthol reagent [31, 33].

### Whole-Genome Sequencing

The gDNA of strain MBLB3053^T^ was extracted according to the method described above. Whole-genome sequencing was performed using a Pacific Biosciences RS II instrument equipped with P6-C4 chemistry. The de novo genome assembly was conducted using Flye assembler 2.7, employing the default parameters within PacBio SMRT Analysis v. 2.3.0 [34]. The confirmation of identity for strain MBLB3053^T^ involved cross-referencing the 16S rRNA gene sequence obtained through conventional Sanger sequencing with that derived from whole-genome sequencing, as detailed previously. The complete genome sequence of the strain MBLB3053^T^ has been deposited in the NCBI database under the accession number NZ_JAVQLW000000000. To validate the integrity of each sequence and identify potential genome assembly contaminants, the ContEst16S algorithm was utilized (https://www.ezbiocloud.net/tools/contest16s) [35].

### Comparative Genomic Analysis

A comprehensive comparative genomic analysis was undertaken to enhance our understanding of the genomic landscape of the novel strain. The genomes of the type strains closely related to strain MBLB3053^T^ were obtained from the NCBI database (http://www.ncbi.nlm.nih.gov/genome/), namely *P. aestuariivivens* (WMIE01000000), *P. aminovorans* (LN832559), *P. contaminans* (CP020612), *P. denitrificans* (FNEA01000000), *P. halophilus* (JRKN01000000), *P. laeviglucosivorans* (NZ_FXTK01000000), *P. litorisediminis* (WMIG01000000), *P. marinus* (VJYZ01000000), and *P. versutus* (QUMX01000000). To evaluate genomic relatedness, the Ortho-average nucleotide identity (OrthoANI) between the MBLB3053^T^ strain and the aforementioned closely related species was calculated using the OAT software v. 0.93.1 [36]. In silico DNA–DNA hybridization (*is*DDH) values were determined using the Genome-to-Genome Distance Calculator (GGDC 2.1; http://ggdc.dsmz.de/distcalc2.php), employing the recommended formula based on DNA–DNA hybridization to effectively assess genetic similarity between the MBLB3053^T^ strain and its closely related taxa within the genus *Paracoccus* [37, 38]. For intergenomic comparisons and assessment of the relatedness of strain MBLB3053^T^ to other members of the genus *Paracoccus*, the Type Strain Genome Server (TYGS) (http://tygs.dsmz.de/) was utilized. A phylogenomic tree was constructed to visualize evolutionary relationships using the FastME 2.1.4 algorithm, with branch support provided through SPR post-processing utilizing Genome Blast Distance Phylogeny (GBDP) distances. The numbers positioned above the branches represent pseudo-bootstrap support values calculated based on 100 replications [39].

### Genome and Secondary Metabolite Annotation

A thorough in silico genome annotation of strain MBLB3053^T^ was conducted utilizing the Rapid Annotations Using Subsystems Technology (RAST) server (http://rast.nmpdr.org/) [40, 41]. This critical annotation process serves to attribute functions to genes, providing insights into the potential biological roles of various genetic elements within the genome. To further elucidate the genetic landscape, predicted homologous genes were systematically categorized into functional groups based on the Clusters of Orthologous Groups (COG) categories using EggNOG v5.0 [42, 43]. Identification of secondary metabolite biosynthetic gene clusters (BGCs) within the genome was accomplished using antiSMASH 6.0, with strict detection criteria employed to ensure accuracy [44]. Various algorithms, including KnownClusterBlast, ClusterBlast, SubClusterBlast, ActiveSiteFinder, and RREFinder, were applied to extract additional features and annotations from these clusters. To confirm the reliability of gene function assignments, NCBI BLASTp (https://blast.ncbi.nlm.nih.gov/) was used, thereby corroborating the findings from the annotation process.

### Pigment Extraction and Analysis

Strain MBLB3053^T^ was cultivated in R2A broth and the broth culture underwent centrifugation at 12,000 ×*g* for 5 min to harvest bacterial cells. Subsequently, the cells were subjected to treatment with 5 ml of acetone/methanol (7:3, v/v). To ensure comprehensive decolorization, the pellet–organic solvent suspension underwent incubation at 4°C and 200 rpm overnight in the dark. Following the confirmation of proper cell decolorization, the upper organic layers of the extracts were consolidated through centrifugation at 12,000 ×*g* for 15 min. The gathered extracts were evaporated at 40°C using a Clever Evaporator C1 (BioChromato, USA) and then redissolved in methanol/dichloromethane (1:1, v/v). To obtain purified carotenoid samples, the extracts were filtered using a hydrophobic polytetrafluoroethylene (PTFE) syringe filter with a pore size of 0.5 μm (Advantech Co., Ltd., Japan). The maximum absorbance spectra of the crude carotenoid extracts were analyzed using a YL9100 Plus HPLC system equipped with a YL9160 photodiode array (PDA) detector (YOUNGIN Chromass, Anyang, Korea). A YMC Carotenoid C30 reverse-phase (RP) column (dimensions: 5 μm, 250 mm length, and 4.6 mm inner diameter; YMC Co., Japan) was employed for separations. Each injection (20 μl) underwent separation using a gradient eluent composed of methanol/water (92:8, v/v) and 10 mM ammonium acetate as solvent A. Solvent B, consisting of 100% tert-butyl methyl ether, served as the mobile phase. The elution of carotenoid extracts occurred over 20 min, initially with a gradient of 90% solvent A and 10% solvent B. A linear gradient ensued, reaching 83%solvent A and 17% solvent B at the 29 min mark, followed by a sharp linear gradient for 35 min to attain 30%solvent A and 70% solvent B. Ultimately, a linear gradient was initiated to achieve 25% solvent A and 75% solvent B at the 42 min mark. Reversion to the original conditions was maintained for over 60 min to achieve balance. The profiles were continuously recorded using a PDA detector operating between 200 and 600 nm, maintaining a flow rate of 1 mL/min throughout the HPLC analysis [45]. The standard material all-*trans*-astaxanthin was purchased from Sigma-Aldrich (https://www.sigmaaldrich.com/KR/ko/product/sigma/sml0982) to identify the astaxanthin production of strain MBLB3053^T^.

### Antioxidant Potential of Carotenoid Extract

The antioxidant activity of carotenoid extract from strain MBLB3053^T^ was assessed through the free-radical scavenging potential of extracted carotenoids using 0.1 mM 1,1-diphenyl-2-picryl-hydrazine (DPPH, Sigma-Aldrich, USA) dissolved in methanol [46]. Various concentrations (0.44, 0.87, 1.75, 3.5, and 7 ppm) of methanolic carotenoid extract (20 μl) were reacted with 180 μl of 0.1 mM methanolic DPPH (1:9, v/v) and incubated at room temperature for 30 min in dark. Subsequently, absorbances were recorded using a UV/Vis spectrophotometer at 517 nm. The percentage of DPPH scavenging effect was determined using the formula: % DPPH scavenging effect = [(A_0_ - A_1_) / A_0_] × 100, where A_0_ represents the absorbance of the control (20 μl of solvent with 180 μl of DPPH solution) and A1 denotes the absorbance of the sample (20 μl of carotenoid extract with 180 μl of DPPH solution). To confirm the antioxidant properties of the carotenoid extract from MBLB3053^T^, the commercial antioxidants butylated hydroxytoluene (BHT, Duksan, Seoul, Korea), 6-hydroxy-2,5,7,8-tetramethylchromane-2-carboxylic acid (Trolox, Sigma-Aldrich, USA), and synthetic astaxanthin (Sigma-Aldrich) were tested simultaneously, and analyzed by ANOVA with Tukey’s multiple comparisons test (p* < 0.05, p** < 0.01, and p*** < 0.001, bars represent mean with standard deviation).

## Results and Discussion

### 16S rRNA Gene Phylogeny

**Fig. 1:**
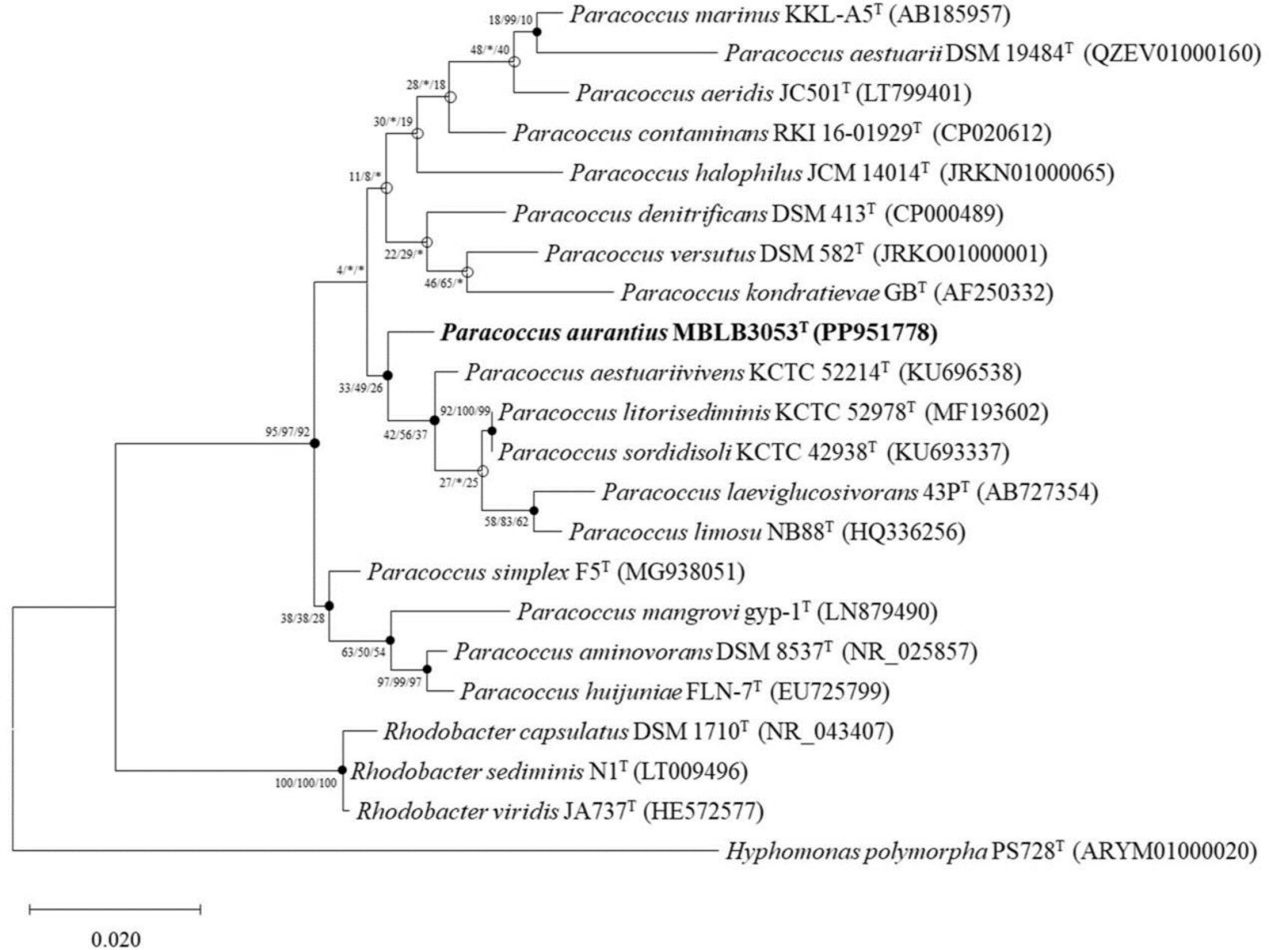
Maximum likelihood (ML) phylogenetic tree of strain MBLB3053^T^ based on the 16S rRNA gene sequences. Phylogenetic tree was constructed with closely related species of the genus *Paracoccus*. The closed circles represent nodes recovered by both the neighbor-joining (NJ) and maximum parsimony (MP) algorithms; the open circles represent nodes recovered by either NJ or MP. The numbers on the nodes indicate the bootstrap values (>70%) calculated using the ML/ NJ/MP probabilities. Bar, 0.02 accumulated changes per nucleotide, respectively.

The sequence of a partial 16S rRNA gene fragment (1,321 bp) from strain MBLB3053^T^ was obtained through Sanger sequencing as mentioned above. In the subsequent phylogenetic analysis, strain MBLB3053^T^ exhibited under 98.5% similarities with other closely related species of the genus *Paracoccus* (Table S1). The most closely relatives and similarities were identified as *P. aestuariivivens* KCTC 52214^T^ (98.5%), *P. litorisediminis* KCTC 52978^T^ (98.3%), and *P. sordidisoli* KCTC 42938^T^ (98.3%). The 16S rRNA gene phylogeny showed that the closest clustered species to strain MBLB3053^T^ was *P. aestuariivivens* KCTC 52214^T^ by the ML and MP algorithms (Fig. 1). According to the phylogenetic analysis based on 16S rRNA gene sequences, strain MBLB3053^T^ was predicted to belong to the genus *Paracoccus*. However, the overall low bootstrap values in the phylogenetic tree suggest a lack of support for the inferred relationship between strain MBLB3053^T^ and other species of the genus *Paracoccus*. Therefore, genome-based comparisons were necessary.

### Phenotypic Characteristics

**Fig. 2:**
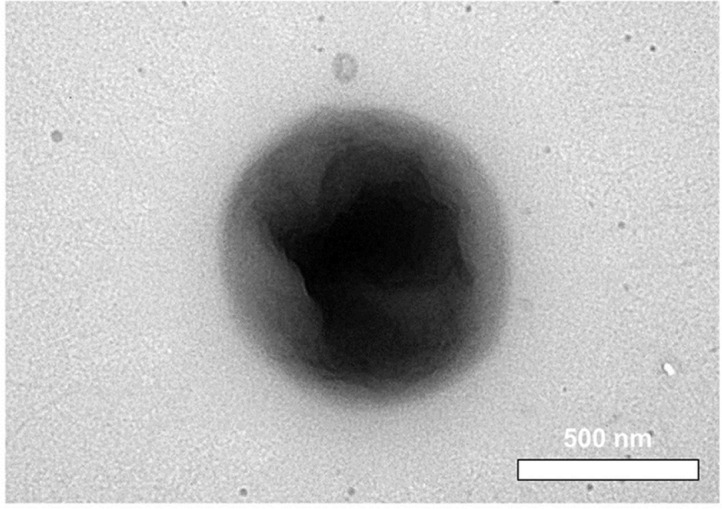
Negatively stained transmission electron micrograph of strain MBLB3053^T^. The strain was cultivated at 30ºC for 2 days in R2A plate. Bar: 500 nm.

Cells of the novel strain MBLB3053^T^ were gram-negative, non-motile, and coccoid with a diameter of 0.7–1.1 μm × 0.7–1.0 μm as revealed by TEM (Fig. 2). Colonies grown on R2A plate for 2 days were orange in color, circular, smooth, and convex. Growth of strain MBLB3053^T^ occurred at 10–40°C (optimum, 30°C), pH 6.0–10.0 (optimum, pH 7.0), and in 0–7.0% (w/v) NaCl concentrations (optimum, 2%). Positive reaction was detected in the catalase and oxidase test. Strain MBLB3053^T^ did not hydrolyze casein, starch, CMC, Tween 20, 40, and 80. H2S was not produced and growth did not occur under anaerobic condition on R2A plate. The strain was susceptible to ampicillin, cephalothin, erythromycin, gentamicin, kanamycin, lincomycin, neomycin, norfloxacin, novobiocin, streptomycin, and tetracycline, but not to penicillin G. Several phenotypic properties were found to be shared between strain MBLB3053^T^ and closely related *Paracoccus* species, such as some enzymatic properties, including urease, β-glucosidase, alkaline phosphatase, esterase (C4), leucine arylamidase, acid phosphatase, naphthol-AS-BI-phosphohydrolase, and α-glucosidase. In addition, the utilization of carbon sources, including D-arabinose, L-arabinose, D-ribose, D-xylose, D-galactose, D-glucose, D-fructose, D-mannose, D-lyxose, D-fucose, and L-fucose, was found to be shared. However, strain MBLB3053^T^ can be differentiated from its closely related species based on several phenotypic properties, such as colony color and utilization of 5-ketoglutanate (Table 1). These results indicate that strain MBLB3053^T^ is a distinct species from other members of the genus *Paracoccus*. Other detailed physiological and biochemical characteristics of strain MBLB3053^T^ were given in Table 1.

### Chemotaxonomic Characteristics

The cellular fatty acid profiles of strain MBLB3053^T^ and the three related species of *P. aestuariivivens* KCTC 52214^T^, *P. litorisediminis* KCTC 52978^T^, and *P. sordidisoli* KCTC 42938^T^ were compared in Table 2. The major fatty acid (more than 10% in fatty acids profiles) was summed feature 8, which included C_18:1_ ω7c/ω6c. The major fatty acid of strain MBLB3053^T^ was similar to those of the three related species. However, there were differences in the proportions of some fatty acids and the presence of C_18:0_ iso, C_10:0_ 3OH, and C_16:1_ ω7c/ω6c, although these existed in the three phylogenetically related species. The predominant isoprenoid quinone of strain MBLB3053^T^ was ubiquinone-10 (Q-10), which was also the case in those related species of the genus *Paracoccus* [15-17]. The major polar lipids detected in strain MBLB3053^T^ were diphosphatidylglycerol (DPG), phosphatidylcholine (PC), and phosphatidylglycerol (PG), and aminoglycolipid (AGL) (Fig. S1). The one phospholipid, two phosphoglycolipids, one aminolipid, one aminoglycolipid, one glycolipid, and three unidentified lipids were also present in minor amounts. These chemotaxonomic properties of strain MBLB3053^T^ were typical of other strains found in the genus *Paracoccus*.

### General Genome Features and Authenticity

The genome sequence of strain MBLB3053^T^ comprised 8 contigs (4,603,921 bp), and the total G + C content of DNA was 62.5 mol%. Strain MBLB3053^T^ was predicted to harbor 4,471 genes, including 4,336 coding genes, 74 RNA genes, and 61 pseudogenes. The number of rRNAs, tRNAs, and ncRNAs were 12, 59, and 3, respectively. More detailed general genomic features of the strain MBLB3053^T^ are presented in Table 3. The comparison of 16S rRNA gene sequences obtained from whole-genome sequencing and conventional Sanger sequencing confirmed 100% similarity. The authenticity of the genome was verified as the genome sequences were not contaminated by comparing four 16S rRNA gene fragments.

### Genome-Based Phylogeny

**Fig. 3:**
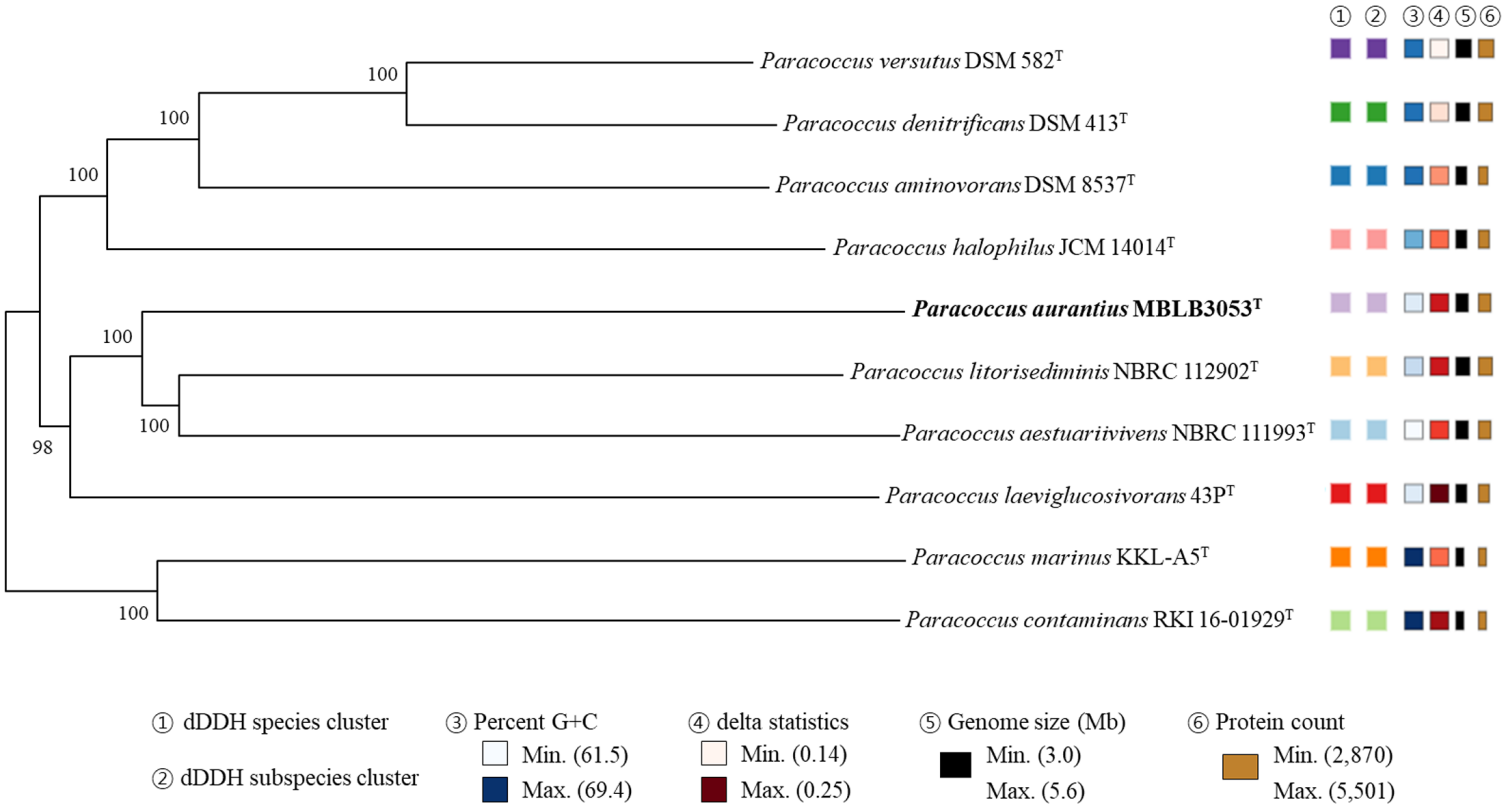
Phylogenomic tree based on TYGS results showing the relationship between strain MBLB3053^T^ with related type strains of the genus *Paracoccus*. The whole-genome sequence-based tree was generated with FastME 2.1.6.1 from GBDP distances calculated from genome sequences. The different colors of the dDDH species and subspecies clusters indicate that the strains in the phylogenomic tree were different. The branch lengths are scaled in terms of GBDP distance formula d5. The numbers above branches are GBDP pseudo-bootstrap support values of >60% from 100 replications, with an average branch support of 99.7%.

The OrthoANI values computed by comparison between strain MBLB3053^T^ and other species of the genus *Paracoccus* ranged from 74.0 to 80.4% (Table 4). In particular, strain MBLB3053^T^ had 79.5% nucleotide identity with *P. aestuariivivens* KCTC 52214^T^, which showed high similarity in 16S rRNA gene sequence comparison, and 80.4% with *P. litorisediminis* KCTC 52978^T^. In addition, the *is*DDH values obtained by comparison between strain MBL3053T and other species of the genus *Paracoccus* did not exceed 23.4% (Table 4). According to the suggested cut-off values of OrthoANI and *is*DDH for species delineation (less than 95‒96%, and 70%, respectively), the calculated values based on the genome analysis results indicated that strain MBLB3053^T^ was distinguished from other previously reported *Paracoccus* species [36, 38, 47]. Additionally, the phylogenomic tree showing intergenomic relatedness between the isolate and closely related species in the genus *Paracoccus* revealed that ten species, including strain MBLB3053^T^, showed different genome features and were not involved in equivalent species or subspecies clustering (Fig. 3) [40]. Overall analysis including 16S rRNA gene similarities and the genome comparison data indices indicated that strain MBLB3053^T^ could be proposed as a novel species of the genus *Paracoccus*.

### Functional Gene Annotations

According to the subsystem information of the RAST analysis, strain MBLB3053^T^ had a total of 4,630 coding genes. Among them, 1,157 genes were annotated in subsystem feature counts. Of these, amino acids and derivatives (326) and protein metabolism (194) were the most abundant subsystems category distribution in strain MBLB3053^T^ (Table S2). In addition, COG analysis showed that a total of 4,504 genes were present in strain MBLB3053^T^, and 3,001 genes (74.0%) associated with the 19 general COG functional categories were classified as coding orthologs. Amino acid transport and metabolism (E: 522, 12.88%) and transcription (K: 325, 8.02%) were the most abundant orthologs (Table S3). Based on the RAST and COG analysis, strain MBLB3053^T^ was predicted with various genes present that were related to protein and amino acid.

The nine BGCs for secondary metabolites production (T1PKS, hserlactone, redox-cofactor, thioamide-NRP, terpene, NRPS, ectoine, NRP-metallophore, and tioamitides) were found in the genome of strain MBLB3053^T^ (Table S4). Among them, a terpene gene type was predicted to produce carotenoid, and terpene BGCs shared 87.0% cluster similarity with *Paracoccus* sp. N81106. Specifically, we focused on terpene BGC reports on carotenoid biosynthesis in *Paracoccus* species. Several genes involved in the carotenoid biosynthesis pathway in strain MBLB3053^T^ were located within the cluster shown in Table S5. The gene cluster related to carotenoid biosynthesis pathway consisted of isopentenyl diphosphate isomerase (*idi*) polyprenyl synthetase (*crtE*), phytoene synthase (*crtB*), phytoene desaturase (*crtI*), lycopene cyclase (*crtY*), β-carotene ketolase (*crtW*), and β-carotene hydroxylase (*crtZ*). These are confirmed to be identical to the previously reported astaxanthin biosynthetic pathway in the *Paracoccus* spp [50, 51]. The ectoine BGC shared 83.0% cluster similarity with *Methylomicrobium alcaliphilum* 20Z^T^. The BGC features of strain MBLB3053^T^ were thoroughly characterized using genome analysis and secondary metabolite annotation, which also shed light on the strain's putative biological roles and capacity to create secondary metabolites.

### Identification of Carotenoid

**Fig. 4:**
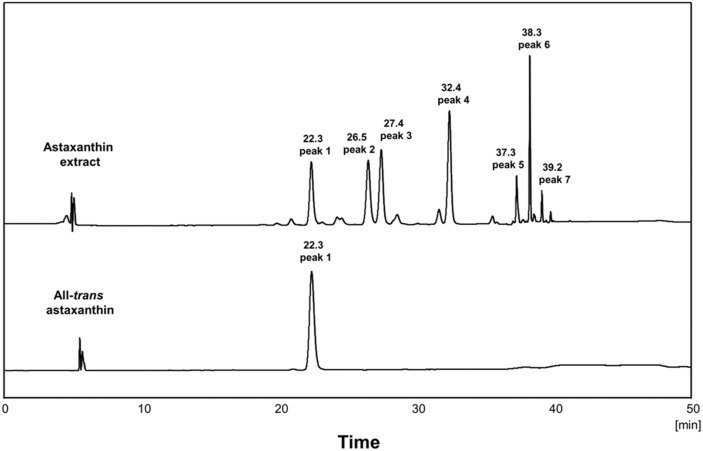
Chromatographic analysis of carotenoid extracted from strain MBLB3053^T^ compared to all-*trans*astaxanthin.

The orange carotenoid was extracted from the novel strain MBLB3053^T^ and subjected to HPLC-PDA analysis, which revealed more than seven peaks exhibited as major carotenoids (Fig. 4). The chromatographic peaks eluting at 22.3, 26.5, 27.4, 32.4, 37.3, 38.3, and 39.2 min had an identifiable spectrum, respectively. Specifically, the UV-Vis absorption spectrum showed that each peak shared a distinct signature carotenoid maximum absorption spectrum at 468–472 nm (Fig. S2). According to the above results, most of the carotenoid extracts derived from strain MBLB3053^T^ were astaxanthin. Compared with the standard material, the peak eluting at 22.3 min was all-*trans*-astaxanthin. Although additional analysis is needed, other chromatograms were predicted to be astaxanthin isomers, and strain MBLB3053^T^ was confirmed to produce various structures of astaxanthin. The production of astaxanthin is not applicable to all members of the genus *Paracoccus*. Comprehensive extraction and analysis of three closely related strains (*P. aestuariivivens* KCTC 52214^T^, *P. litorisediminis* KCTC 52978^T^, and *P. sordidisoli* KCTC 42938^T^) to MBLB3053^T^ revealed few peaks on the chromatogram, confirming the distinctive absence of astaxanthin production in these strains (Fig. S3). Notably, astaxanthin production emerges as a unique characteristic among the species closely related with strain MBLB3053^T^.

### Antioxidant Activity of Astaxanthin Extract

**Fig. 5:**
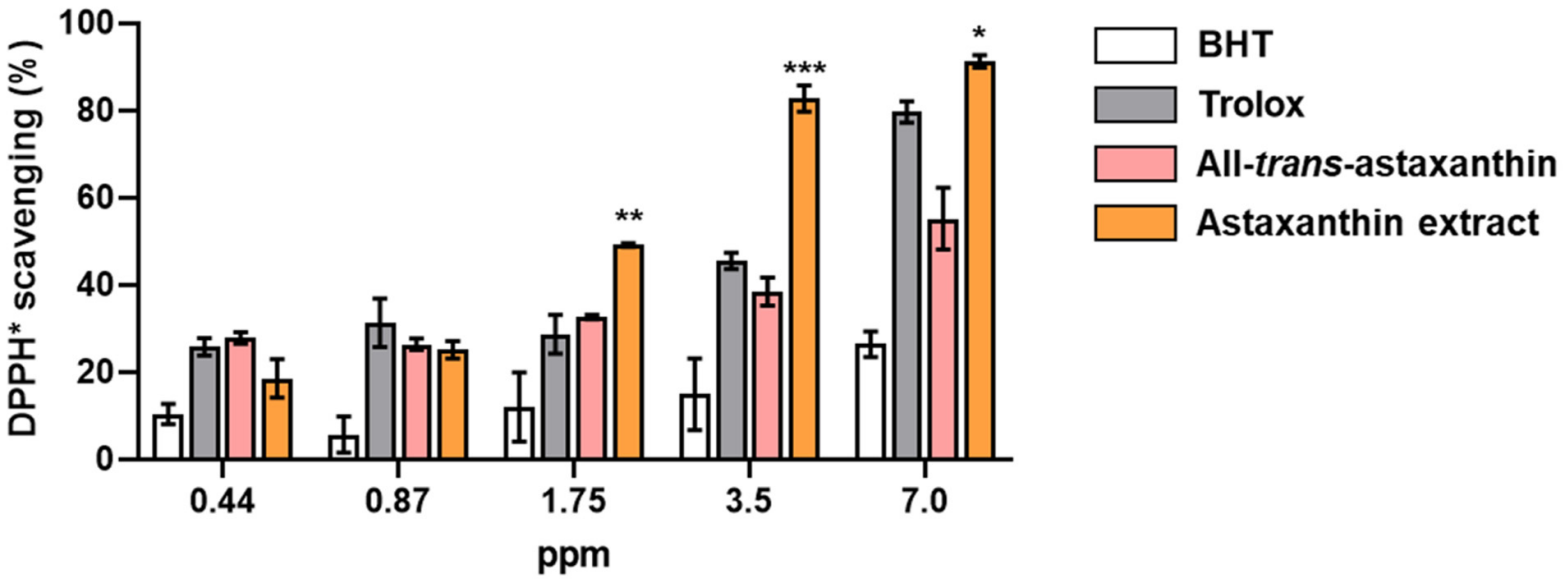
In vitro antioxidant activities of astaxanthin extract from strain MBLB3053^T^ compared with other antioxidants. DPPH-scavenging activities were evaluated in the presence of different concentrations (*p < 0.05, **p < 0.01, and ***p < 0.001; bars represent mean ± SD (*n* = 3)). BHT, butylated hydroxytoluene; Trolox, 6-Hydroxy-2,5,7,8- tetramethylchromane-2-carboxylic acid.

The DPPH free-radical scavenging activity of astaxanthin extract derived from strain MBLB3053^T^ was investigated. The DPPH-scavenging activity increased according to each antioxidant concentration. Astaxanthin extract from strain MBLB3053^T^ exhibited significantly higher DPPH radical-scavenging activity as shown in Fig. 5. At a low concentration below 0.87 ppm, MBLB3053^T^-derived astaxanthin extract showed lower effectiveness than other antioxidants (18.6 ± 4.4 and 25.1 ± 2.0%, respectively). When the concentrations were above 1.75 ppm, astaxanthin extract showed significantly higher than BHT, Trolox, and all-*trans*-astaxanthin. At 1.75, 3.5 and 7 ppm, astaxanthin extract showed DPPH radical-scavenging activities of 49.1 ± 0.4, 82.7 ± 3.0, and 91.2 ± 1.5%, respectively. These scavenging values were significantly different from Trolox, which showed the highest radical-scavenging activities (28.7 ± 4.5% at 1.75 ppm, 45.5 ± 1.9% at 3.5 ppm, and 79.6 ± 2.5% at 7 ppm) among the comparison groups. Especially, astaxanthin extract from strain MBLB3053^T^ had a higher antioxidant effect than the same concentration of synthetic astaxanthin at most concentrations. A previous report demonstrated that in vitro antioxidant activity of the *cis* isomers of astaxanthin exhibits a higher performance compared to the all-*trans* isomer [52]. These results suggest that the *Paracoccus* carotenoid extracted from novel species (especially astaxanthin) could be considered as an attractive natural antioxidant bioresource.

### Description of *Paracoccus aurantius* sp. nov.

***Paracoccus aurantius* sp. nov. (au.ran’ti.us. N.L. masc. adj. aurantius, orange, pertaining to colony color).** Cells are gram-negative, non-motile, and coccoid with a diameter of 0.7–1.1μm × 0.7–1.0μm. The colonies are circular, smooth, convex, and orange in color when grown on R2A agar at 30°C for 2 days. Growth is observed at a temperature range of 10–40°C (optimum, 30°C), pH 6.0–10.0 (optimum, pH 7.0), in 0–7.0% (w/v) NaCl concentrations (optimum, 2%). Casein, starch, CMC, Tween 20, 40, and 80 are not hydrolyzed, while the catalase and oxidase tests are positive. H_2_S is not produced and growth does not occur under anaerobic condition on R2A plate. In API 20NE test, reduction of nitrate to nitrite, urease, and β-glucosidase activities are positive, but indole production, glucose fermentation, arginine dihydrolyase, protease (gelatin), and β-galactosidase are negative. In the API ZYM test, alkaline phosphatase, esterase (C4), leucine arylamidase, acid phosphatase, naphtol-AS-BI-phosphohydrolase, and α-glucosidase are positive, but esterase (C8), lipase (C14), valine arylamidase, cystine arylamidase, trypsin, α-chymotrypsin, α-galactosidase, β-galactosidase, β-glucronidase, β-glucosidase, N-acetyl-β-glucosaminidase, α-mannosidase, and α-fucosidase are negative. The major fatty acids (>10%) include summed feature 8 (C_18:1_ ω7c and/or C_18:1_ ω6c; 66.75%). The predominant isoprenoid quinone is ubiquinone-10 (Q-10), and the major polar lipids are DPG, PC, PG, and AGL. The genome size of the type strain is 4.6 Mb with a G+C value of 62.5 mol%. The NCBI GenBank accession number for the 16S rRNA gene sequence is PP951778, and the NCBI accession number for the whole-genome assembly is NZ_JAVQLW000000000. The type strain, MBLB3053^T^ (=KCTC 8269^T^ = JCM 36634^T^), was isolated from coastal waters of Dokdo (Seodo) Island.

## Supplemental Materials

Supplementary data for this paper are available on-line only at http://jmb.or.kr.



## Figures and Tables

**Table 1:** Differential morphological and physiological characteristics of strain *P. aurantius* MBLB3053^T^ with closely related species of the genus *Paracoccus*.

Characteristics	1	2	3	4	5
Morphology*	Coccoid	Short rods, Coccoid	Coccoid	Short rods, Coccoid	Rods
Colony color*	Light orange	Yellowish white	Yellowish white	Creamy white	White to light yellow^d^
Temperature range for growth (°C)	10–40 (30)*	15–45 (30)^a^	10–40 (30)^b^	8–40 (28)^c^	10–40 (20–30)^d^
pH range for growth	6.0–10.0 (7.0)*	5.0–10.5 (6.5–7.5)^a^	5.5–9.5 (7.0–8.0)^b^	5.0–11.0 (6.0–9.0)^c^	6.0–8.0 (7.0)^d^
NaCl tolerance (%)	0–7 (2)*	0–8 (2)^a^	0–5 (2)^b^	0–6 (0–4)^c^	0–7 (2)^d^
API 20 NE*					
Reduction of nitrates	+	+	+	-	+^d^
API ZYM*					
Valine arylamidase	-	+	-	-	+^d^
Cystine arylamidase	-	-	+	+	+^d^
API 50CH*					
Glycerol	-	+	+	+	+^e^
Erythritol	-	-	-	+	+^e^
L-xylose	-	-	-	+	-^e^
D-adonitol	-	-	+	-	+^e^
D-fructose	+	+	-	+	+^e^
L-sorbose	-	-	+	-	-^e^
L-rhamnose	-	+	+	+	+^e^
Inositol	-	-	+	+	+^e^
D-sorbitol	-	-	+	-	+^e^
Xylitol	-	+	+	+	+^e^
D-turanose	-	-	-	+	+^e^
D-tagatose	-	-	+	+	-^e^
D-arabitol	-	+	+	+	+^e^
5-ketogluconate	+	-	-	-	-^e^
DNA G+C contents (mol%)	62.5	62.0^a^	64.1^b^	67.2^c^	66.8^e^

1. *P. aurantius* MBLB3053^T^; 2. *P. aestuariivivens* KCTC 52214^T^ [15]; 3. *P. litorisediminis* KCTC 52978^T^ [16]; 4. *P. sordidisoli* KCTC 42938^T^ [17]; 5. *P. denitrificans* DSM 413^T^ [48, 49].

Data from *this study., ^a^Park *et al*. [15]; ^b^Park *et al*. [16]; ^c^Singh *et al*. [17]; ^d^Chen *et al*. [48]; ^e^Wang *et al*. [49]

**Table 2:** Cellular fatty acids profiles of strain MBLB3053^T^ with closely related species of the genus *Paracoccus*.

Fatty acid	1	2	3	4
Saturated				
C_14:0_	-	TR	-	-
C_16:0_	8.13	5.73	5.91	5.78
C_17:0_	4.62	-	TR	TR
C_18:0_	3.12	1.33	1.76	2.26
Unsaturated				
C_17:1_ ω7c	-	TR	1.11	-
C_20:1_ ω7c	-	-	-	TR
Branched-chain fatty acid				
C_10:0_ iso	1.33	-	-	-
C_18:0_ iso	-	TR	1.71	1.11
C_18:1_ ω7c 11-methyl	4.55	-	3.11	-
Hydroxy fatty acid				
C_10:0_ 3OH	-	4.11	4.04	3.91
CYCLO				
C_19:0_ cyclo ω8c	7.18	2.49	-	-
Other				
C_16:0_ N alcohol	-	TR	-	-
Summed feature*				
1	-	-	-	-
2	4.32	2.18	2.34	2.31
3	-	1.52	1.01	TR
8	66.75	79.94	78.48	82.79

1. *P. aurantius* MBLB3053^T^; 2. *P. aestuariivivens* KCTC 52214^T^; 3. *P. litorisediminis* KCTC 52978^T^; 4. *P. sordidisoli* KCTC 42938^T^.

All data obtained from this study. -. Not detected; TR, Traces (<1.0%).

*Summed features are groups of two or more fatty acids that could not be separated using the MIDI system. Summed feature 1, C_15:1_ iso H/C_13:0_ 3OH; Summed feature 2, C_14:0_ 3OH/C_16:1_ iso I; Summed feature 3, C_16:1_ ω7c/ω6c; 4, C_18:1_ ω7c/ω6c; Summed feature 8, C_18:1_ ω7c/ω6c.

**Table 3:** General characteristics of the genome of strain MBLB3053^T^ with closely related species of the genus *Paracoccus*.

Attribute	1	2	3
Sequencing platforms	PacBio Sequel	Illumina HiSeq	Illumina HiSeq
Assembler	FLYE v. 2.8.3	SOAP denovo v. 2.04	SOAP denovo v. 2.04
Accession number	NZ_JAVQLW000000000	WMIE01000000	WMIG01000000
Genome coverage	2,049.2x	221.0x	188.0 x
Assembly size (bp)	4,603,921	4,560,478	5,370,867
N50 (bp)	2,850,522	244,239	220,239
G + C content (mol%)	62.5	61.4	63.6
Total contigs	8	77	72
Total gene	4,471	4,460	5,273
Pseudo gene	61	102	88
Total CDS	4,336	4,300	5,215
RNAs	74	58	58
- rRNA genes (5S, 16S, 23S)	4, 4, 4	1, 1, 1	1, 1, 1
- tRNA	59	52	52
- ncRNAs	3	3	3

1. *P. aurantius* MBLB3053^T^; 2. *P. aestuariivivens* NBRC 111993T; 3. *P. litorisediminis* NBRC 112902T

**Table 4:** OrthoANI, *is*DDH values, and model confidence interval between strain MBLB3053^T^ and closely related species of the genus *Paracoccus*.

Query genome	Reference genome	OrthoANI value (%)	IsDDH value (%)	Model interval (%)
MBLB3053^T^	*Paracoccus litorisediminis* NBRC 112902^T^	80.4	23.4	[21.1 - 25.9]
	*Paracoccus aestuariivivens* NBRC 111993^T^	79.5	22.9	[20.6 - 25.3]
	*Paracoccus aminovorans* DSM 8537^T^	78.2	21.7	[19.4 - 24.1]
	*Paracoccus versutus* DSM 582^T^	78.1	22.1	[19.9 - 24.6]
	*Paracoccus denitrificans* DSM 413^T^	78.1	21.8	[19.5 - 24.2]
	*Paracoccus halophilus* JCM 14014^T^	77.5	20.9	[18.7 - 23.3]
	*Paracoccus laeviglucosivorans* 43P^T^	77.7	20.9	[18.7 - 23.4]
	*Paracoccus marinus* KKL-A5^T^	74.0	20.0	[17.8 - 22.4]
	*Paracoccus contaminans* RKI 16-01929^T^	74.0	19.9	[17.7 - 22.3]
