# Supplementary material for: Carotenoid-Producing Paracoccus aurantius sp. nov., Isolated from the West Coast of Dokdo Island, Republic of Korea
Source: J Microbiol Biotechnol. 2024 Aug 9;34(10):2012–22. doi: 10.4014/jmb.2404.04053 (PMC11540602; doi:10.4014/jmb.2404.04053)
Supplement: Supplementary file 1 [file jmb-34-10-2012-supple.pdf]

J. Microbiol. Biotechnol. 2024. 34(10): 2012-2022
https://doi.org/10.4014/jmb.2404.04053

Supplementary Tables and Figures

Carotenoid-Producing *Paracoccus aurantius* sp. nov., Isolated from the West-Coast on Dokdo Island, Republic of Korea

Chi Young Hwang[1], Eui-Sang Cho[1,2], Eun Hee Bae[3], Dong-Hyun Jung[3,4], and Myung-Ji Seo[1,5,6*]

[1]Department of Bioengineering and Nano-Bioengineering, Incheon National University, Incheon 22012, Republic of Korea

[2]Biotechnology Institute, University of Minnesota, St. Paul, MN 55108, USA

[3]Climate Change and Environmental Biology Research Division, National Institute of Biological Resources, Incheon 22689, Republic of Korea

[4]Division of Food and Nutrition, Chonnam National University, Gwangju 61186, Republic of Korea

[5]Division of Bioengineering, Incheon National University, Incheon 22012, Republic of Korea

[6]Research Center for Bio Materials & Process Development, Incheon National University, Incheon 22012, Republic of Korea

Table S1.

16S rRNA gene similarities between *Paracoccus aurantius* MBLB3053^T and closely related type strains of the genus *Paracoccus*.

Taxon	Accession no.	Similarity (%)
Paracoccus aestuariivivens GHD-30^T	KU696538	98.49
Paracoccus litorisediminis GHD-05^T	MF193602	98.26
Paracoccus sordidisoli LP91^T	KU693337	98.26
Paracoccus shanxieyensis DK398^T	MN044987	98.18
Paracoccus simplex F5^T	MG938051	97.73
Paracoccus laeviglucosivorans 43P^T	AB727354	97.58
Paracoccus limosus NB88^T	HQ336256	97.43
Paracoccus contaminans RKI 16-01929^T	CP020612	97.34
Paracoccus aminovorans DSM 8537^T	jgi.1055360	97.20
Paracoccus mangrovi gyp-1^T	LN879490	97.20
Paracoccus versutus DSM 582^T	JRKO01000001	97.19
Paracoccus marinus KKL-A5^T	AB185957	97.12
Paracoccus kondratievae GB^T	AF250332	97.05

Table S2.

Subsystem category distribution in *Paracoccus aurantius* MBLB3053^{T} genome.

Subsystem feature	Count
Cofactors, vitamins, prosthetic groups, pigments	155
Cell wall and capsule	22
Virulence, disease and defense	32
Potassium metabolism	3
Miscellaneous	25
Membrane transport	81
Iron acquisition and metabolism	12
RNA metabolism	33
Nucleosides and nucleotides	109
Protein metabolism	194
Motility and chemotaxis	5
Regulation and cell signaling	17
Secondary metabolism	5
DNA metabolism	71
Fatty acids, lipids, and isoprenoids	72
Nitrogen metabolism	14
Dormancy and sporulation	1
Respiration	107
Stress response	72
Metabolism of aromatic compounds	49
Amino acids and derivatives	326
Sulfur metabolism	10
Phosphorus metabolism	26
Carbohydrates	185
**Total**	**1,157**

Table S3.

COG categories of coding proteins in strain MBLB3053^{T} with closely related species of the genus *Paracoccus*. 1, *P. aurantius* MBLB3053^{T}; 2, *P. aestuariivivens* NBRC 111993^{T}; 3, *P. litorisediminis* NBRC 112902^{T}.

Code	Description	1		2		3	
		Count	%	Count	%	Count	%
J	Translation, ribosomal structure, and biogenesis	177	4.37	182	4.49	184	4.00
K	Transcription	325	8.02	327	8.07	387	8.41
L	Replication, recombination and repair	164	4.05	181	4.47	198	4.30
D	Cell cycle control, cell division, chromosome partitioning	46	1.13	47	1.16	56	1.22
V	Defense mechanisms	45	1.11	40	0.99	46	1.00
T	Signal transduction mechanisms	92	2.27	105	2.59	113	2.46
M	Cell wall/membrane/envelope biogenesis	198	4.88	192	4.74	198	4.30
N	Cell motility	39	0.96	36	0.89	41	0.89
U	Intracellular trafficking, secretion, and vesicular transport	48	1.18	49	1.21	39	0.85
O	Posttranslational modification, protein turnover, chaperones	132	3.26	124	3.06	141	3.06
C	Energy production and conversion	255	6.29	239	5.90	290	6.30
G	Carbohydrate transport and metabolism	226	5.57	264	6.52	282	6.13
E	Amino acid transport and metabolism	522	12.88	507	12.51	583	12.67
F	Nucleotide transport and metabolism	90	2.22	88	2.17	98	2.13
H	Coenzyme transport and metabolism	122	3.01	114	2.81	129	2.80
I	Lipid transport and metabolism	164	4.05	167	4.12	169	3.67
P	Inorganic ion transport and metabolism	267	6.59	287	7.09	358	7.78
Q	Secondary metabolites biosynthesis, transport, and catabolism	88	2.17	99	2.44	111	2.41
B	Chromatin structure and biogenesis	2	0.05	1	0.02	2	0.04
S	Function unknown	1,053	25.97	1,003	24.75	1,174	25.51
**Total**		**4,054**	**100.00**	**4,052**	**100.00**	**4,602**	**100.00**

Table S4.

Distribution of BGCs of strain *Paracoccus aurantius* MBLB3053^{T} and similar known pathways with strict detection criteria.

Gene type	Product	Span (nt)	Most similar biosynthetic gene cluster (BGC)	BGC similarity (%)
T1PKS		386,745-433,026		
Hserlactone		448,148-468,738		
Redox-cofacor		1,034,215-1,056,438		
Thioamide-NRP		2,389,560-2,430,819		
Terpene	Carotenoid	2,887,019-2,910,580	*Paracoccus* sp. N81106	87.0
NRPS		2,962,533-3,006,357		
Ectoine	Ectoine	3,415,776-3,426,171	*Methylomicrobium alcaliphilum* 20Z^{T}	83.0
NRP-metallophore		4,157,258-4,182,557		
Thioamitides		4,191,314-4,213,949		

Table S5.

Carotenoid biosynthetic gene organization and homology analysis of strain MBLB3053^T in terpene criteria.

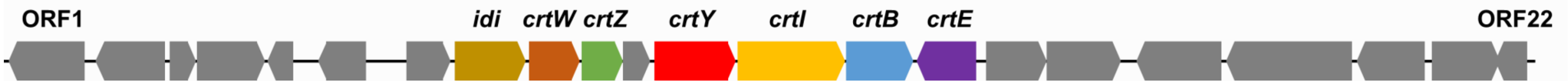

Name	Top hit	Description	E value	Identity (%)
ORF1	WP_134682240	NnrS family protein [*Paracoccus ravus*]	0.0	74.74
ORF2	WP_134682239	NO-inducible flavohemoprotein [*Paracoccus ravus*]	0.0	84.95
ORF3	WP_155095763	Rrf2 family transcriptional regulator [*Paracoccus aestuariivivens*]	3e-80	85.71
ORF4	WP_023849697	HPP family protein [*Ponticoccus alexandrii*]	0.0	69.66
ORF5	WP_217330280	SH3 domain-containing protein [*Paracoccus* sp. Z118]	9e-52	86.96
ORF6	WP_209021323	SOS response-associated peptidase family protein [*Paracoccus ravus*]	1e-122	86.73
ORF7	WP_167626649	TetR/AcrR family transcriptional regulator [*Paracoccus luteus*]	1e-108	75.73
ORF8 (*idi*)	WP_182791876	Type 2 isopentenyl-diphosphate Delta-isomerase [*Paracoccus* sp. MC1854]	0.0	84.66
ORF9 (*crtW*)	WP_217629650	Beta-carotene ketolase [*Paracoccus chinensis*]	5e-142	81.67
ORF10 (*crtZ*)	WP_095640462	Beta-carotene hydroxylase [*Paracoccus salipaludis*]	4e-110	86.86
ORF11		Hypothetical protein		
ORF12 (*crtY*)	WP_095640460	Lycopene beta-cyclase [*Paracoccus salipaludis*]	0.0	78.65
ORF13 (*crtI*)	WP_090753294	Phytoene desaturase [*Paracoccus chinensis*]	0.0	91.18
ORF14 (*crtB*)	WP_090753295	Phytoene/squalene synthase family protein [*Paracoccus chinensis*]	4e-173	85.71
ORF15 (*crtE*)	WP_175558768	Polyprenyl synthetase family protein [*Paracoccus chinensis*]	5e-139	70.95
ORF16	HEV7434598	TPA: ABC transporter ATP-binding protein [*Pseudorhizobium* sp.]	8e-156	71.75
ORF17	WP_142589147	ABC transporter permease [Pseudorhizobium halotolerans]	6e-180	71.14

ORF18	WP_276990317	FAD-binding oxidoreductase [*Lentibacter algarum*]	7e-153	52.55
ORF19	WP_163039024	TRAP transporter permease [*Aliiroseovarius* sp. PrR006]	0.0	68.44
ORF20	RMF33021	TAXI family TRAP transporter solute-binding subunit [*Alphaproteobacteria bacterium*]	5e-169	69.46
ORF21	WP_283780936	LysR substrate-binding domain-containing protein [*Ruegeria spongiae*]	8e-112	51.60
ORF22	WP_206614230	Transposase [*Sinirhodobacter populi*]	1e-23	46.40

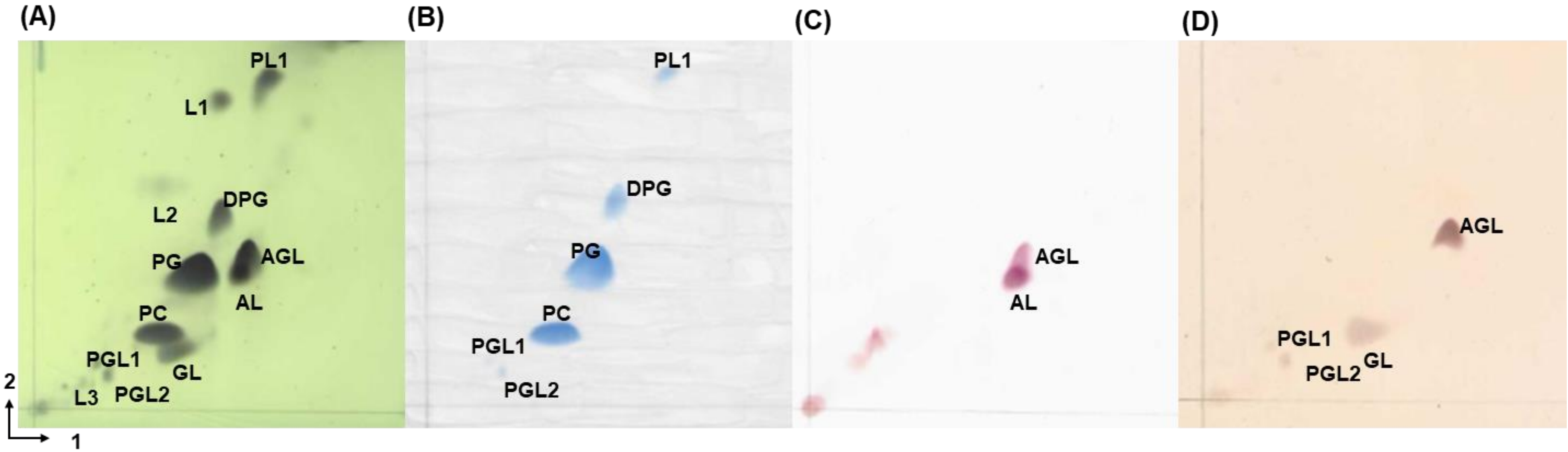

Fig. S1. Thin-layer chromatograms of the polar lipids of strain MBLB3053^{T}. (A) Total lipids; **(B)** Phospholipids; **(C)** Aminolipids; **(D)** Glycolipids. The 1st-D developing agent, chloroform:methanol:water (65:25:4, v/v/v) and the 2nd-D agent, chloroform:acetic acid:methanol:water (80:15:12:4, v/v/v/v). Sulfuric acid-ethanol (1:2, v/v) for total lipids, Zinzade's regent (5% of ethanolic molybdatophosphoric acid) for total phospholipids, ninhydrin for amino-containing lipids. The major polar lipids are DPG, PC, PG, and AGL. PL, phospholipid; PGL, phosphoglycolipid; AL, aminolipid; AGL, aminoglycolipid, L, unidentified lipid.

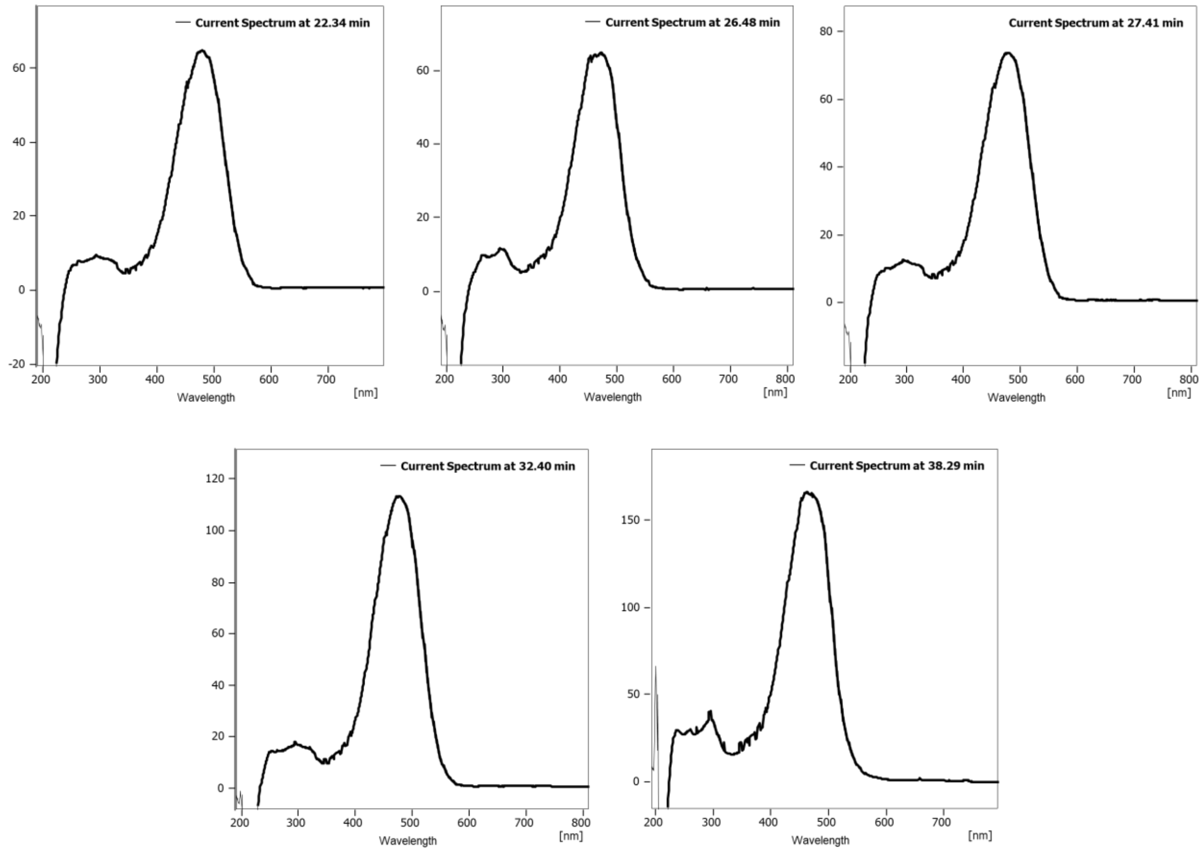

Fig. S2. UV visible maximum absorption spectrum of the orange carotenoid extracted from strain MBLB3053^{T}.

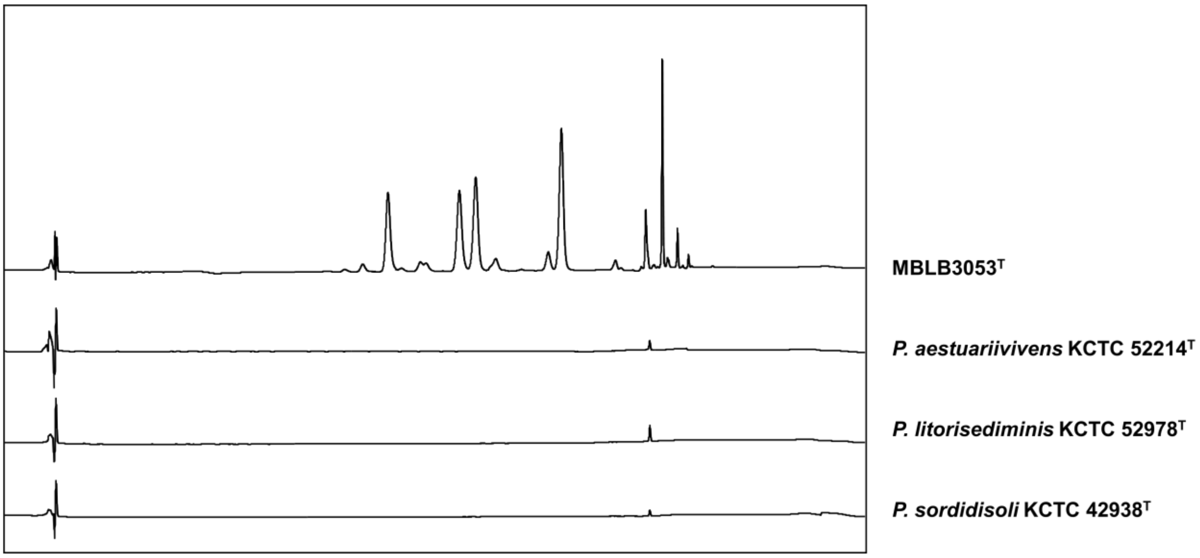

Fig. S3. HPLC analysis chromatograms of carotenoid extract from strain MBLB3053^T and other closely related species (*P. aestuariivivens* KCTC 52214^T, *P. litorisediminis* KCTC 52978^T, and *P. sordidisoli* KCTC 42938^T).
